# Clinical and Preclinical Evidence for Adverse Neurodevelopment after Postnatal Zika Virus Infection

**DOI:** 10.3390/tropicalmed6010010

**Published:** 2021-01-12

**Authors:** Jessica Raper, Ann Chahroudi

**Affiliations:** 1 Yerkes National Primate Research Center, Emory University, Atlanta, GA 30329, USA; jraper@emory.edu; 2 Department of Pediatrics, Emory University School of Medicine, Atlanta, GA 30322, USA; 3 Center for Childhood Infections and Vaccines of Children’s Healthcare of Atlanta and Emory University, Atlanta, GA 30322, USA

**Keywords:** flavivirus, MRI, emotion, social, cognition, rhesus macaque, pediatric, postnatal, neonatal

## Abstract

Although the Zika virus (ZIKV) typically causes mild or no symptoms in adults, during the 2015−2016 outbreak, ZIKV infection in pregnancy resulted in a spectrum of diseases in infants, including birth defects and neurodevelopmental disorders identified in childhood. While intense clinical and basic science research has focused on the neurodevelopmental outcomes of prenatal ZIKV infection, less is known about the consequences of infection during early life. Considering the neurotropism of ZIKV and the rapidly-developing postnatal brain, it is important to understand how infection during infancy may disrupt neurodevelopment. This paper reviews the current knowledge regarding early postnatal ZIKV infection. Emerging clinical evidence supports the hypothesis that ZIKV infection during infancy can result in negative neurologic consequences. However, clinical data regarding postnatal ZIKV infection in children are limited; as such, animal models play an important role in understanding the potential complications of ZIKV infection related to the vulnerable developing brain. Preclinical data provide insight into the potential behavioral, cognitive, and motor domains that clinical studies should examine in pediatric populations exposed to ZIKV during infancy.

## 1. Introduction

Zika virus (ZIKV) is a neurotropic flavivirus that is primarily transmitted by the bite of an *Aedes* genus mosquito, but it has also been transmitted through sexual contact, blood transfusions, organ transplantation, and from mother to fetus during gestation [1,2]. Although vaccine clinical trials are currently under investigation [3], there are currently no licensed vaccines to prevent or targeted therapeutics to treat ZIKV infection. The main preventative measure is the avoidance of mosquito bites, from which it is difficult to ensure 100% protection. 

Despite being first isolated in 1947, ZIKV was relatively unknown to the public for 70 years because it typically resulted in asymptomatic or mild symptoms in the majority of adults [4,5]. However, during the 2015−2016 outbreak in Brazil, it was discovered that ZIKV infection during pregnancy could result in birth defects, leading to the declaration of a global health emergency [6,7,8,9,10,11,12,13]. ZIKV spread rapidly across the Americas, and infections have now been reported in 91 countries and territories [13,14]. While ZIKV incidence no longer constitutes a current epidemic, continued infections occur, and ZIKV has adapted to persistent endemic transmission [15]. In fact, in a recent serologic study, 9% of children aged 1–4 years were ZIKV seropositive in Indonesia, highlighting the widespread transmission in young children living in endemic areas [16]. ZIKV is transmitted primarily by the bite of an *Aedes* genus mosquito, but also via sex, blood transfusions, organ transplantation, and from mother to fetus. 

The passage of ZIKV into the brain, and its ability to induce pathological changes have been reported since the late 1950s [17,18,19]. During the 2015−2016 outbreak, it was discovered that ZIKV could infect neural stem cells and neural progenitor cells, causing their eventual apoptosis [20]. Data from fetal human brain development suggests that radial glia and intermediate progenitors are particularly susceptible to ZIKV infection [21,22,23]. Recent evidence also suggests an interaction between ZIKV-infected microglia and altered neural progenitor cell differentiation and proliferation [24]. Congenital infection with ZIKV occurs throughout gestation, with resultant microcephaly and other brain malformations [21,25,26,27] that are thought to be the consequence of the ZIKV infection of neural progenitor cells, as well as the activation of innate immune responses [28]. Congenital ZIKV syndrome is a pattern of birth defects that includes severe microcephaly, the thinning of the cerebral cortex with subcortical calcifications, macular scarring and retinal mottling, congenital contractures, and hypertonicity [29]. Infants with congenital ZIKV syndrome can develop seizures, hearing and vision problems, feeding difficulties, and gross motor abnormalities [30]. While microcephaly is probably the most salient feature of congenital ZIKV syndrome, it does not occur in all cases of prenatal exposure. In fact, a prospective study of 216 toddlers with prenatal ZIKV exposure reported microcephaly in only eight (3.7%) of the cases [31]. Although some studies report that head circumference at birth corresponds with abnormal posture and motor skills during infancy [32,33], a recent report found no correlation between head size at birth and gross motor function at 24 months of age [34]. In fact, Nielsen-Saines and colleagues found that, despite few cases of microcephaly, one third of children with prenatal ZIKV exposure had below-average cognitive, language or motor scores on the Bayley-III evaluation [31]. Thus, one cannot assume that infants born without microcephaly or obvious signs of congenital ZIKV syndrome will experience normal development. Language, motor, and cognitive functions gradually develop over years in early childhood, which coincides with the prolonged maturation of the brain areas that are important for these skills [35,36]. Considering this protracted development, reports of infants prenatally infected with ZIKV exhibiting the postnatal onset of microcephaly, neurologic dysfunction, and neurodevelopmental abnormalities [10,12,37,38] further highlight the potential of ZIKV to cause ongoing damage after birth. 

## 2. Clinical Evidence of Postnatal Zika Virus Infection

Postnatally, the brain matures exponentially, particularly in the temporal, prefrontal and parietal regions that are important for social, emotional, and executive functions, including learning, attention, and memory throughout the first two years of age in humans [35,39,40,41,42,43,44,45,46]. This highly dynamic period of postnatal brain development presents a time of great vulnerability. Prolonged synaptic proliferation and neuronal maturation during postnatal development not only contribute to learning and periods of plasticity, but also allow for environmental factors to affect the maturation of both the brain and behavior [40,47,48]. Considering the neurotropism of ZIKV, can infection during infancy disrupt this crucial period of neurodevelopment? 

The evidence shows that infants and children can acquire ZIKV infection postnatally, through mosquito bites and, possibly, breast milk [49]. Children account for 10−31% of ZIKV infections in various studies [50,51,52]. However, the data on ZIKV in children are still sparse; many studies include a wide age range in their pediatric population (1 month to 18 years), and few include significant numbers of children infected with ZIKV at <1 year of age [53,54,55,56,57,58,59,60]. Acute neurologic complications of ZIKV infection in children have been described, including Guillian-Barre Syndrome, polyneuropathy, encephalitis, demyelinating disease, and inflammatory diseases of the central nervous system (CNS) [58,59,61]. A meta-analysis of pediatric ZIKV infection found that these cases are primarily mild, and most present with a fever and rash [56], but severe neurologic complications and death have also been reported [59,62,63,64]. 

Beyond the acute infection period, there have been few studies of neurodevelopment following postnatal ZIKV infection. A notable recent study of the neurologic outcomes of ZIKV included six children who were infected postnatally, one of whom was 10 months old at the time of infection and developed severe CNS involvement [65]. Additionally, a prospective study of 60 children with postnatal ZIKV infection between 1 and 12 months of age found that 15% had adverse neurologic, hearing or eye examinations at 20−30 months of age [66]. An additional 12.8% received an alert score in the hearing domain. For those without abnormal neurologic, eye, or hearing outcomes, there was also a positive correlation between their age at ZIKV infection and their percentile score on the Personal–Social domain, as assessed by the Escala Abreviada de Desarrollo (EAD-1), meaning that the infants who were infected later performed better. These data suggest that the neurotropism of ZIKV can lead to adverse neurodevelopmental consequences for vulnerable young brains, but the full extent of this impact is still largely unknown. There is, at present, no compelling evidence to suggest either for or against the severity of symptoms during acute infection being predictive of neurodevelopmental outcomes. As with congenital ZIKV infection in which adverse neurodevelopment has been reported in children without overt birth defects, one might speculate that mild or asymptomatic postnatal ZIKV infection in children has the potential to be associated with subsequent neurodevelopmental deficits.

## 3. Preclinical Models of Postnatal Zika Virus Infection

Animal models of postnatal ZIKV infection have the potential to be highly informative, considering the paucity of studies in children. Postnatally ZIKV-infected neonatal mice demonstrate extensive apoptotic degeneration in several brain regions, including the hippocampus, with activation being followed by the fragmentation of the microglia [67]. Impairments in motor and cognitive functions were also found in mice infected with ZIKV postnatally [68]. However, ZIKV infection in postnatal 3-day old mice most closely resembles the second or third trimester of gestation in humans [69]. In contrast, nonhuman primate (NHP) development more faithfully parallels humans, but on a condensed time frame, with an 1:4 ratio of monkey to human lifespan [70,71]. Like humans, NHPs have complex cognitive ability and primarily use their visual system to detect and respond to social cues from other group members, and their brain develops in a temporal and anatomical manner that is similar to humans. For these reasons, NHPs represent a highly translational animal model for the investigation of the neurodevelopmental impact of postnatal ZIKV infection in humans. 

During the 2015−2016 ZIKV epidemic, we gathered a team of experts in pediatric infectious disease, flaviviruses, and neuroscience in order to investigate the consequences of postnatal ZIKV infection in infant rhesus macaques [72,73]. Six infant rhesus macaques were infected with a Puerto Rican strain of ZIKV (PRVABC59 at 10^5^ plaque-forming units, subcutaneously) at 5 weeks of life, which is equivalent to a 4−5 month old human. Viremia peaked at 2–3 days post-infection, and was cleared from the blood by 7 days. Despite the quick viral clearance and the lack of rash or fever in the ZIKV-infected infant macaques, we demonstrated ZIKV dissemination to the central and peripheral nervous systems. ZIKV RNA was found in the lymph nodes and spleen at the peak of the viremia, with ZIKV RNA also being detected in the neurons and myeloid lineage cells in the central and peripheral nervous systems by day 14–15 after infection. The histologic examination of the brain at 2 weeks post-infection revealed pathological features such as inflammation, reactive astrocytes, gliosis, axonal injury and apoptosis, which have also been reported in congenital ZIKV infection. Upon the confirmation that ZIKV disseminates to the CNS postnatally, our next goal was to investigate whether this resulted in neurodevelopmental consequences that could be tracked in vivo. 

**Figure 1:**
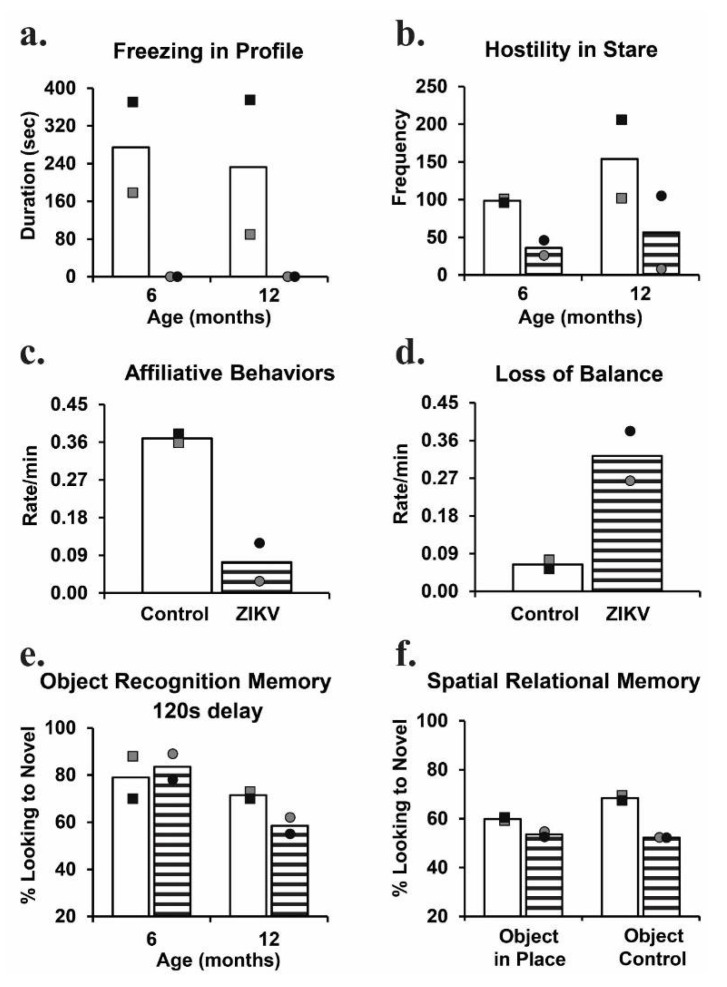
Behavioral consequences of postnatal ZIKV infection. This illustrates the changes in the emotional reactivity (**a**,**b**), social behavior (**c**), motor functions (**d**), and cognition (**e**,**f**) of the ZIKV-infected infant macques (dashed bars with circles) and controls (open bars with squares). Reproduced with modifications from Mavigner, et al. (2018) [72] and Raper et al. (2020) [73].

Social, emotional, motor, and cognitive skills develop across the first years of life, which correspond with postnatal brain development [74,75,76,77]. We followed two ZIKV-infect infant macaques, and two age-, sex-, and rearing-matched controls for 12 months post-infection in order to investigate how ZIKV dissemination may have impacted the development of their brains and behavior. The ability to modulate one’s emotional response based on the salience of a threat in the environment is an essential skill, which can be impacted by early brain insult or environmental factors [78,79,80,81,82]. In order to investigate whether postnatal ZIKV infection impacted emotional behavior development, we utilized the Human Intruder Paradigm to test the behavioral reactivity at 6 and 12 months of age [72]. This paradigm is a robust test modeled after the Stranger Approach Task (also called the Strange Situation Task), which is used to assess behavioral inhibition and anxiety in children [83,84]. Specifically, the paradigm examines the ability of the animal to change its behavioral reaction based on the presence and gaze direction of an unfamiliar person. When faced with the mild threat of the person’s profile (no eye contact/Profile condition) the uninfected controls exhibited the species-typical response of increased freezing, while the ZIKV-infected infant macaques did not (Figure 1a). Normally-developing infant macaques exhibit increased hostility behaviors when faced with the more salient threat of the person’s direct gaze (Stare condition) [85,86]. This species-typical behavioral response to the increasing level of threat was not present in the ZIKV-infected infant macaques compared to the uninfected controls (Figure 1b). Overall, these data suggest that postnatal ZIKV infection disrupted the emotional behavior development of the macaques, resulting in an inability to appropriately modulate responses based on the level of the threat presented. 

The ability to appropriately perceive and respond to behavioral cues from others in our environment is also an essential skill for human and nonhuman primates. At 12 months of age, we conducted 4.5 h of social behavior observations of ZIKV-infected and uninfected control monkeys in a large social-play cage [73]. Unlike the uninfected controls, the ZIKV-infected macaques took longer to habituate to this novel environment. This aversion for novelty may be indicative of increased behavioral inhibition or anxiety after postnatal ZIKV infection. Despite the pairs of animals being highly familiar cage mates, the ZIKV-infected macaques spent less time together and engaged in less prosocial behaviors compared to the uninfected controls (Figure 1c). Our finding parallels that of Pacheco and colleagues [66], such that 20–30-month old children with postnatal ZIKV infection during early infancy scored more poorly on the Personal–Social domains of the EAD-1. Poor social skills on the EAD-1, including difficulties recognizing and interacting with others, are similar to the increased time apart and decreased affiliative behaviors exhibited by the highly familiar pair of ZIKV-infected macaques. The large play cage (390 cubic feet) provides the monkeys with more space to run, jump, swing, and play, and also creates opportunities to exhibit uncoordinated movements resulting in losses of balance. The ZIKV-infected and control macaques spent similar amounts of time playing, but the ZIKV-infected macaque infants tended to exhibit more losses of balance compared to the uninfected controls (Figure 1d). Impaired motor function has also been reported in several cases of postnatal ZIKV infection in children, such that children infected at younger ages (infancy and toddler) exhibit gait difficulties, while older kids (adolescent) appear to recover motor abilities within a month post-infection [87,88,89,90,91]. Observational results suggest that ZIKV infection during infancy has a long-term impact on emotional, social, and gross motor function. 

A progressive developmental pattern is also evident in learning and memory functions, making them susceptible to environmental influences and early brain insult [92,93,94,95]. The visual paired comparison (VPC) task measures recognition memory by measuring the unique tendency of human and nonhuman primates to direct their attention to novel stimuli in their environment. The VPC task can be administered to nonverbal subjects with immature motor skills, making it an invaluable tool for the measurement of memory in young children and NHPs. Starting at 6 months of age, we examined simple object recognition memory in postnatal ZIKV-infected and uninfected control macaques [72,73]. Specifically, Object-VPC measures delay-dependent object recognition memory using familiarization to a picture of a single colored object and delays varying from 10 to 120 s. The groups did not differ in their performance at 6 months of age, but at 12 months, the ZIKV-infected macaques exhibited an impairment at the longest delay compared to the uninfected controls (Figure 1e). While simple object recognition can be demonstrated in early infancy, spatial relational memory matures between 12 and 18 months in rhesus macaques. At 12 months of age, we used Object-in-Place and Object-Control VPC tasks [73], which require the ability to learn not only the identities of a set of co-presented items, but also to learn and remember their spatial arrangement. We found that postnatal ZIKV-infected macaques were unable to perform with an increasing memory load, indicating an impairment in visuospatial processes (Figure 1f). These data demonstrate that ZIKV infection during infancy can negatively impact recognition memory function. Memory impairment suggests that postnatal ZIKV infection affects the development of the brain areas that are important for visual learning and memory function. The lack of observed impairment at the earlier age we evaluated is reflective of the studies demonstrating that early hippocampal insults result in cognitive impairments that emerge later in development [96,97], meaning that longer clinical follow up may be required in order to detect the impact of postnatal ZIKV infection on learning and memory function. 

**Figure 2:**
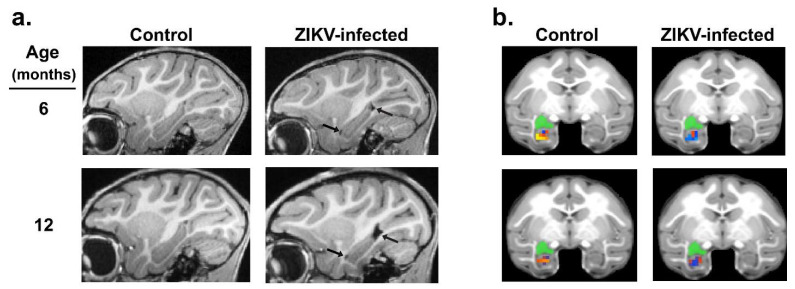
Consequences of postnatal ZIKV infection on neurodevelopment. Magnetic resonance imaging (MRI) in infant macaques illustrates the impact of early ZIKV exposure on hippocampal growth (**a**) (black arrows point to increased lateral ventricle volume, also indicating decreased hippocampal volume) and resting-state functional connectivity (**b**) between the left amygdala (green seed) and hippocampus at 6 and 12 months of age. Reproduced with modifications from Mavigner, et al. (2018) [72] and Raper et al. (2020) [73].

Considering the alterations in the emotional, social, motor and cognitive functioning detected in ZIKV-infected macaques, we used in vivo magnetic resonance imaging (MRI) to determine the potential structural and functional alterations in the brain regions that control these functions. Structural and resting-state functional MRI scans were taken at 3, 6, and 12 months of age in ZIKV-infected and uninfected control macaques [72,73]. Although no differences were detected in the overall total brain or intracranial volume at any age, MRI scans revealed that the growth of specific brain regions was impacted in postnatal ZIKV infection. Compared to the uninfected controls, the ZIKV-infected macaques exhibited increased lateral ventricle size, but decreased hippocampal, amygdala, and putamen volume across the first year of life (Figure 2a). Importantly, these structural changes in the brain corresponded with functional differences, such that the ZIKV-infected macaques had weaker functional connectivity between the left amygdala and hippocampus compared to the controls (Figure 2b). Together, these neuroimaging results indicate that ZIKV infection during infancy can lead to alterations in brain development and function that are detected as early as 3 months of age, and persist through to 12 months of age.

Neurohistopathology was conducted in order to investigate the long-term impact of ZIKV infection during infancy. Unlike the neurohistopathology conducted 14 days post-infection, no significant lesions, apoptosis, or gliosis were detected in the spinal cord, cauda equina, or in the brain of ZIKV-infected macaques at 12 month of age [73]. However, neuropathological examination revealed ventriculomegaly as well as neuropil and perivascular calcification in the putamen of the ZIKV-infected macaques, confirming the findings detected with the in vivo MRI. Lastly, underdeveloped dendritic branching of immature amygdala neurons was detected in the ZIKV-infected macaques compared to the uninfected controls. The neurohistopathology findings demonstrate that postnatal ZIKV infection can result in brain lesions that impact the long-term growth and functioning of the brain in the absence of ongoing inflammatory changes.

There are limitations to the preclinical data provided by the NHP model of postnatal ZIKV infection. First, studies in NHPs often have limited sample size due to the constraints of working with a scarce resource, as well as budgetary considerations. A second and related limitation is that sex as a biologic variable has not yet been addressed (in our work, only female subjects were used to investigate the long-term effects of postnatal ZIKV infection, precluding the examination of potential sex differences in ZIKV infection, as has been reported in mice [98]). Third, the age of infection has thus far been limited to early infancy, limiting our knowledge of how infection during later periods may either spare or impair neurodevelopment. Fourth, for consistency and comparison to prior NHP studies, a single strain of ZIKV (PRVABC59) was used for the postnatal challenge. This strain is closely related to isolates from Brazil [99] but likely does not fully capture the spectrum of ZIKV infections. In addition to these limitations, several important knowledge gaps remain regarding postnatal ZIKV infection in human infants and children. Many of these (definitive rates of adverse neurodevelopment, specific neurologic tests that should be used in clinical follow up, the ways in which acute symptomatology may predict long term outcomes) have been described above. A further key missing set of data that may be addressed by the preclinical NHP model of postnatal ZIKV is whether adverse outcomes can be prevented by targeted treatment during acute infection. These limitations and gaps in knowledge could be addressed with future NHP studies that include larger sample sizes, both sexes, multiple ages at infection, the use of different strains of ZIKV, and promising antiviral or anti-inflammatory interventions.

## 4. Conclusions

Our studies add to the growing literature showing that viral infection during infancy, including cytomegalovirus and HIV, can negatively affect brain development, leading to long-term neurological damage and cognitive impairment [100,101,102,103]. The highly dynamic period of postnatal brain development presents a time of great vulnerability. Few studies have examined ZIKV infection during infancy; as such, the extent of the damage that may be caused by ZIKV infection during this sensitive postnatal period is largely unknown. A further gap in our understanding stems from a lack of long-term follow up studies for children who contract ZIKV in early infancy. Considering that early life damage to temporal lobe structures results in cognitive impairments that are not fully revealed until later in childhood [96], translational studies in animal models will provide important insight regarding the specific developmental processes that may need to be monitored in infants and children that have been exposed to ZIKV postnatally. Other than our own work, we were not able to find any studies designed to interrogate the neuropathogenesis of postnatal ZIKV infection and the resultant developmental consequences using NHP models, although two reports describe postnatal ZIKV susceptibility in NHPs that were also exposed prenatally to ZIKV [104,105]. The features of congenital ZIKV syndrome have been recapitulated after fetal exposure in NHPs, with visual, hearing, neurobehavioral, and brain changes having been observed [106]. Given the spectrum of clinical manifestations following postnatal ZIKV infection that have been described, and given that some, but not all, children develop neurological symptoms and impaired neurodevelopment, it is paramount that future research efforts be dedicated to the understanding of these disparate outcomes. This research will likely entail adequately-powered NHP studies in which the virologic, immunologic, and neurologic parameters can be measured and manipulated in order to determine the resultant range of phenotypes. Furthermore, large scale clinical studies to track neurodevelopment after postnatal ZIKV infection are needed. Our data suggest that clinical follow up studies should investigate longitudinal changes in emotional regulation, and social, gross motor, and cognitive function in children with postnatal ZIKV infection acquired in infancy. 

## Data Availability

Not applicable.
